# PORTAL: Pilot study on the safety and tolerance of preoperative melatonin application in patients undergoing major liver resection: a double-blind randomized placebo-controlled trial

**DOI:** 10.1186/1471-2482-8-2

**Published:** 2008-01-23

**Authors:** Peter Schemmer, Arash Nickkholgh, Heinz Schneider, Michael Sobirey, Markus Weigand, Moritz Koch, Jürgen Weitz, Markus W Büchler

**Affiliations:** 1 Department of General Surgery, Ruprecht-Karls University, Heidelberg, Germany; 2 Department of Anesthesiology, Ruprecht-Karls University, Heidelberg, Germany; 3 HealthEcon AG, Basel, Switzerland; 4 Nutri~fit GmbH & Co KG, Mühlen, Germany

## Abstract

**Background:**

Major surgical procedures facilitate systemic endotoxinemia and formation of free radicals with subsequent inflammatory changes that can influence the postoperative course. Experimental data suggest that preoperative supraphysiological doses of melatonin, a potent immuno-modulator and antioxidant, would decrease postoperative infectious and non-infectious complications induced by major abdominal surgery.

**Methods/Design:**

A randomized controlled double blind single center clinical trial with two study arms comprising a total of 40 patients has been designed to assess the effects of a single preoperative dose of melatonin before major liver resection. Primary endpoints include the determination of safety and tolerance of the regimen as well as clinical parameters reflecting pathophysiological functions of the liver. Furthermore, data on clinical outcome (infectious and non-infectious complications) will be collected as secondary endpoints to allow a power calculation for a randomized clinical trial aiming at clinical efficacy.

**Discussion:**

Based on experimental data, this ongoing clinical trial represents an advanced element of the research chain from bench to bedside in order to reach the highest level of evidence-based clinical facts to determine if melatonin can improve the general outcome after liver resection.

**Trial Registration:**

EudraCT200600530815

## Background

Surgical injury triggers a proinflammatory cascade of hormonal, immunologic, and cellular responses collectively known as acute phase response to minimize damage and to hasten healing and recovery as John Hunter once described it as "both the disposition and the means of cure [1]." However, the consequences of this sophisticated sequence of events are not always advantageous for the surgical patient due to the varying magnitude of the response and the severity of the injury. Tissue ischemia and the subsequent reperfusion, particularly in visceral organs, as well as loss of intra- and extravascular volume due to hemorrhage, burn injury or extracorporeal circulation can result in oxidative stress, in both surgical and distant sites, microvascular leakage, platelet aggregation, leukocyte-endothelium adhesion, and depression of humoral and cell-mediated immunity that subsequently lead to proinflammatory changes [2]. Systemic endotoxinemia is accentuated due to the leakage of aerobic and anaerobic microflora from the gastrointestinal tract through microvascular damage resulting from ischemia/reperfusion injury, altered immunity secondary to the inflammatory cascade, visceral ischemia and necrosis, hemorrhage and hypotension [3]. Under these circumstances the occurrence of transient endotoxinemia and formation of free radicals is almost certain with both infectious and non-infectious postoperative complications as possible consequences. Surgical infections, identified as a major limiting factor to the postoperative clinical recovery, do occur in spite of preventive treatment with antibiotics immediately before or during surgical treatment and represent a major challenge especially when more complex and high-risk operations are performed in more debilitated patients, and in the face of increasing antibiotic resistance.

### Melatonin

Melatonin (N-acetyl-5-methoxytryptamine) is a hormonal product primarily produced and secreted by the pineal gland, although it has been detected in other tissues as well [4]. Melatonin is involved in the circadian rhythm and the sleep-wake cycle of vertebrates. In addition to its main function of sleep induction, melatonin is also known for its immunomodulating activities as well as for its antioxidative effects [5-7]. Melatonin is the most powerful endogenous free radical scavenger known at present. It not only directly neutralizes a number of free radicals and reactive oxygen species, but also stimulates several antioxidative enzymes which increase its efficiency as antioxidant.

In terms of direct free radical scavenging, melatonin interacts with the highly toxic hydroxyl radical with a rate constant to that of other highly efficient hydroxyl radical scavengers. Additionally, melatonin reportedly neutralizes hydrogen peroxide, singlet oxygen, peroxynitrite anion, nitric oxide and hypochlorous acid and stimulates antioxidant enzymes such as superoxide dismutase, glutathione peroxidase and glutathione reductase. Melatonin can be widely used as a protective agent against a wide variety of processes and agents that damage tissues via free radical and reactive oxygen mechanisms. The highest concentration of melatonin has been found in the hepatobiliary system [8] with high-affinity binding sites in hepatocyte nuclei [9].

### Pharmacokinetics

The antioxidative effects of melatonin require concentrations that are much higher than endogenously produced plasma concentrations (peak plasma levels of approximately 100 pg/ml) [10]. Exogenous oral doses of melatonin >0.3 mg produce already supraphysiological levels in humans [11]. Orally administered melatonin is subject to extensive first-pass metabolism in the liver, and absolute bioavailability is reported to be approximately 15%, although highly variable [12,13]. Melatonin has an elimination half-life (t_1/2_) of 30–45 min [14-17] which is extended to approximately 100 min in cirrhotic patients [15]. In humans, the principal metabolite of endogenous as well as exogenous melatonin is 6-sulphatoxy melatonin [18].

Toxicity of melatonin is remarkably low. It was not possible to determine DL50 for melatonin since the highest possible does (800 mg/kg BW; limitation imposed by the solubility of the compound) failed to induce mortality in mice [19]. When melatonin was given to rats on gestation days 6–19 in very large doses (50–200 mg/kg BW, the doses equal to ~17.5 – 70 g/day) the maternal toxicity NOAEL (No Observed Adverse Effect Load) and LOAEL (Lowest observed adverse effect level) were 100 and 200 mg/kg/day, respectively, and the developmental toxicity NOAEL was >200 mg/kg BW [20]. No significant adverse effects were observed following administration of high doses (3 – 6.6 g/day) to eleven individuals for 15 to 35 days [21].

In the United States, the Food and Drug Administration (FDA) has designated melatonin as an orphan product sold as a dietetic complement with the only therapeutic indication in circadian rhythm disturbances in blind patients [22]. In most European countries this hormone is considered as a scientifically non-evaluated drug, therefore its sale is not allowed [4].

### Trial rationale

Experimental data suggest that melatonin applied preoperatively would be suitable for prevention or prophylactic treatment of postoperative infectious and non-infectious complications induced by major surgical interventions. Exogenous, intragastrically applied melatonin as well as endogenously generated melatonin has been shown to promote healing of gastric ulcers in rats [23]. This antiulcer activity has been attributed to the ability of melatonin to stimulate enzymes that protect against oxidative stress [24,25]. Besides, orally-administered melatonin has been found efficient in reducing negative parameters of cholestasis and provided a hepatoprotective effect against the liver injury secondary to acute ligation of the biliary duct in rats [26,27]. Most recently we observed that rats gavaged with melatonin 2 h before warm ischemia/partial liver resection survive significantly longer than appropriate controls (unpublished data). Enzymes (transaminases) indicative for liver damage were also significantly reduced after preoperative melatonin following warm ischemia in both rats and pigs in a dose dependent manner (unpublished data). In clinical setting, melatonin has been shown to enhance neutrophil apoptosis after partial hepatectomy causing liver ischemia and reperfusion [28], thus eliminating the consequent active inflammatory response due to surgical injury [29]. Most recently, data from a randomized clinical trial has shown intravenous melatonin to decrease oxidative stress status and proinflammatory cytokines in human neonates undergoing surgery for congenital malformations [30]. However, the best administration route, the duration of treatment, the effective dose of melatonin, its exact role in the pathophysiology of surgical diseases, its pharmacological interactions and untoward side effects, as well as its long-term safety has not been fully established yet. Thus, this study has been designed to meet the need for a larger randomized controlled trial to confirm the suggested benefits of melatonin with the consideration of the current knowledge of its immunomodulatory effects.

## Methods/Design

After the positive vote of the ethics committee of the Faculty of Medicine, Ruprecht-Karls University of Heidelberg, Germany, enrollment of the study subjects has been started in June 2007 in this randomized controlled double blind single center clinical trial as a pilot study on the safety and tolerance of preoperative melatonin application in patients undergoing major liver resection. Patients are considered for recruitment to the study according to inclusion and exclusion criteria (Table 1). The duration of the trial for each subject is expected to be 9 days, i.e. 1 day before operation (day -1), the day of operation (day 0) and the first 7 days following the operative procedure (postoperative days 1–7). Considering a recruitment rate up to 5 cases per month, the overall duration of the trial is expected to last approximately 8–10 months. A drop-out rate of 10% (due to non-compliance, intolerance, premature discontinuation, lost to follow-up, etc) will increase the total duration of trial to about 12 months. The actual overall duration of recruitment may vary due to the availability and consent rate of patients undergoing major liver resection.

**Table 1:** Criteria of inclusion and exclusion of patients.

**Inclusion criteria**	**Exclusion criteria**
patients meeting all of the following criteria are considered for inclusion in the study:	patients with any of the following will not be included in the trial:
- scheduled for elective major partial liver resection of ≥3 liver segments for liver neoplasms	- patients <18 and >90 years
- written informed consent	- patients <50 and >120 kg
- adequate contraception is required for women with childbearing potential	- patients with severe, organ-specific disorders (e.g., liver or renal failure, acute pancreatitis)
	- patients undergoing emergency procedures
	- patients with expected intubation problems
	- pregnancy and/or lactation
	- history of hypersensitivity to the investigational product or to any drug with similar chemical structure or to any compound present in the pharmaceutical form of the investigational product
	- simultaneous participation in another clinical trial
	- mental condition rendering the subject incapable of understanding the nature, scope, and consequences of the trial
	No subject will be enrolled in this study more than once.

Before operation demographic data, medical history, physical examination, diagnosis, therapy, as well as comorbidities and concomitant medications are documented on day -1. Furthermore, routine laboratory measurements and substrate monitoring are carried out and documented (Table 2). On the day of surgery and following intubation for general anaesthesia, 500 ml milk containing test (melatonin; Helsinn Chemicals, Biasca, Switzerland) or placebo product (microcrystalline cellulose) (50 mg/kg BW) is administered via a standard gastric tube placed right after intubation of the patient. Clinical laboratory values (including chemistry, hematology, liver laboratory tests, and cytokines) and the substrates are measured on the day of operation and daily throughout the entire postoperative study period of 6 days. Clinical outcome parameters include infectious (pneumonia, wound infection, peritonitis, urinary tract infection, sepsis) as well as non-infectious (bilioma, bile fistula, pleural effusion, bleeding, circulatory insufficiency, renal insufficiency, wound dehiscence, intestinal obstruction/ileus, pulmonary embolism) complications. Sepsis is defined as the presence of at least 2 out of the following 4 criteria: (1) temperature >38°C or <36°C, (2) tachycardia >90/min, (3) respiratory insufficiency (1 out of 3 criteria: respiratory rate >20/min, hyperventilation with PaCO2 <32 mmHg or PaO2 <70 mmHg (spontaneous breathing) or PaO2/FiO2 <175 mmHg (mechanical ventilation), and (4) leukocytosis >12/nl or leukopenia <4/nl. Renal failure is defined according to RIFLE-criteria [31]. The schedule for all study related activities and data collection is listed in Table 2.

**Table 2:** Flow chart, PORTAL study

**Phase**	Pre-OP day^1^	OP day^1^	Post-OP day 1–7^1^
**Study-day**	-1	0	1–7
Medical history	X		
Physical examination	X		
Inclusion/exclusion criteria	X		
Karnowsky index	X		
Patient information	X		
Patient informed consent	X		
Concomitant medication	X		X
Documentation of surgery (OPS code)		X	
**Clinical outcome**			
Bilioma, pleural effusion, pneumonia, wound infection, peritonitis, UTI, sepsis			X
**Safety and Tolerance**			
Adverse events (AE)		X	X
*Liver*			
Integrity: AST, AP, GGT, ALT, LDH, GSH	X	X	X
Synthesis: CE, Chol, TP, PT, PTT albumin, CRP	X	X	X
Elimination: Bilirubin	X	X	X
*Kidney*			
Crea (blood), Na, K, Ca, Cl	X	X	X
Urea-N.	X	X	X
Crea (urine) for Crea-Clearance		X	X
*Substrates, metabolites, cytokines*			
Glucose, TG, lactate, IL-6, melatonin^2^	X	X	X
*Hematology*			
Hb, Hc, Thr, Leuko, Ery	X	X	X

^1 ^sampling at the same time each day, e.g. 8:00, ^2 ^sampling before, 2 h and 4 h after melatonin application

Pre-OP, pre-operative period; OP, operation/day of operation; Post-OP, post-operative period; OPS, "Operations- und Prozedurenschlüssel" [codes for operations and procedures]; UTI, urinary tract infection; AST, aspartate amino-transferase; AP, alkaline phosphatase; GGT, γ-glutamyl transferase; ALT, alanine aminotransferase; LDH, lactate dehydrogenase; GSH, glutathione; CE, choline esterase; Chol, Cholesterol, TP, total protein; PT, prothrombin time; PTT, partial thromboplastin time, CRP, c-reactive protein, Crea, creatinine; Na, natrium; K, kalium; Ca, calcium; Cl, chloride; Urea-N, urea-nitrogen; TG, triglyceride; IL, interlukin; Hb, hemoglobin, Hc, hematocrit, Thr, thrombocyte, Leuko, leukocyte; Ery, erythrocyte.

### Objectives and endpoints

The primary aim of this pilot study is to evaluate safety and tolerance of preoperative application of melatonin in patients undergoing operative procedures for partial liver resection. In addition, clinical parameters reflecting pathophysiological functions of the liver will be determined.

Data on clinical outcome (infectious and non-infectious complications) are collected as secondary endpoints to allow a power calculation for a randomized clinical trial aiming at clinical efficacy (Table 2).

### Randomization and blinding

Randomization (1:1) will be performed by the algorithm programmed in specific computer software and a randomization list will be prepared. Briefly, "blocked randomisation" will be performed using blocks of four that will include equal packages of test product and control product with the order of treatments within a block being randomly permuted, thus ensuring treatment group numbers are evenly balanced at the end of each block. The random number sequence is chosen to start from 1001 and sets an ascending allocation order for the first four subjects. Similarly, treatment type is allocated to the next four patients in the order specified by the next randomly selected permutation. The process is then repeated. The randomization list will be prepared by the institution that is responsible for the packaging of the study products (test product, placebo), i.e. the Pharmacy of the University clinic of Heidelberg, and kept in safe and confidential custody at this institution.

Patients satisfying the enrolment criteria will receive an ascending serial number in the order of their enrolment. Patient numbers for patients who replace drop-outs will be assigned similarly.

Both study products, test (melatonin) and placebo product (microcrystalline cellulose), will be of same form and appearance (white powder in sachets) including the packaging material.

In addition to the trial medication, the clinical trial director (PS) will receive a set of sealed envelopes, one for each randomization number. An identical set of sealed envelopes will be held at the Pharmacy of the University clinic of Heidelberg. These envelopes contain information on the subject's trial medication and are to be opened only under circumstances in which it is medically imperative to know what the subject is receiving.

### Adverse events

An adverse event (AE) is defined as any untoward medical occurrence in a subject administered a pharmaceutical product whether or not related to the investigational product including: new symptoms/medical conditions, new diagnosis, changes of laboratory parameters, intercurrent diseases and accidents, recurrence or worsening of pre-existing co-morbidities, increase of frequency or intensity of episodic diseases. A serious adverse event (SAE) is a life-threatening event resulting in death or significant persistent disability requiring subject hospitalization or prolongation of existing hospitalization. All AEs that occur after the subject has signed the informed consent document to participate in this evaluation of safety and tolerance of preoperative melatonin will be documented on the appropriate pages provided in the electronic Case Report Forms (eCRF). AEs must also be documented in the subject's medical records and must be monitored to determine outcome. The clinical course of the AE will be followed up until its resolution or normalization of changed laboratory parameters or until it has changed to a stable condition. SAEs must be reported using the "Serious Adverse Event" form provided in the eCRF. The initial report must be as complete as possible including details of the event and an assessment of the causal relationship between the event and the trial medication. The clinical trial director must also inform site monitor and data management in all cases.

### Quality assurance

The procedures set out in this trial protocol, pertaining to the conduct, evaluation, and documentation of this trial, are designed to ensure that all persons involved in the trial abide by Good Clinical Practice [32] and the ethical principles described in the current revision of the Declaration of Helsinki [33]. The trial will be carried out in keeping with local legal and regulatory requirements.

### Statistics and data management

All analyses will be carried out on an "intent-to-treat" basis. However, attempts will be made to analyze "per protocol" and "intent-to-treat" populations separately, when statistically appropriate. For the purpose of "per protocol" analysis, two groups of 20 eligible patients completing the protocol will be considered. Drop-out patients are not to be replaced.

The target variables of this trial include laboratory values, outcome parameters and adverse events. The target variables are measured from study day -1 to study day 7. The differences of the laboratory values between study day -1 and 7 are tested with the paired t-test or, in case of uneven distribution, the Wilcoxon-test (2-sided) for within group comparison. The Mann-Whitney U test will be used to detect differences between treatment groups. The following statistical values will be calculated when appropriate: mean, standard deviation, 95%-confidence interval of the mean, minimum, lower quartile, median, upper quartile, maximum, valid number, frequency count, and percentage. The laboratory values will be counted according to their normal ranges (below normal, normal, and above normal). SAS software (Version 8.2) will be used for analysis.

All patient data (clinical and resource use) generated during the study will be recorded on the eCRFs provided by HealthEcon AG, Basel, Switzerland. These forms are specifically designed in Microsoft Office Access (Version: 1.01) to meet the data recording requirements of the clinical study protocol. Corrections of wrong entries will be recorded automatically. In addition, for any correction of data concerning "Adverse Events" or the primary target variable the reason for the correction must be entered. All data will be entered into a central database as recorded.

All data management activities will be done according to the current Standard Operating Procedures (SOPs) of HealthEcon AG.

## Discussion

This clinical trial particularly demonstrates how a scientific hypothesis has been developed from bench to bedside, with classical chain of investigations from early experimental research to the highest level of evidence, a randomized controlled double blind clinical trial. If previous experimental findings are confirmed by the data of this clinical trial, melatonin would improve the overall outcome after major liver resection.

## Competing interests

The author(s) declare that they have no competing interests.

## Authors' contributions

HS, MS, PS and AN participated in the design of the study, developed essential study documents, and formulated the statistical part of the study protocol and the statistical analysis plan. HS, MS and PS performed quality review to assure adherence to current guidelines and laws. MK, JW, MW and MWB supported the design of the study with their knowledge and experience. PS, HS and MS conceived and designed the study based on their own preclinical and clinical results. Further, PS conducts the study as the main investigator and acts as the clinical trial director (Leiter der Klinischen Prüfung (LKP) according to German drug law). HS performs the data management. PS and AN wrote the manuscript. All authors read and approved the final manuscript.

## Pre-publication history

The pre-publication history for this paper can be accessed here:


